# Editorial: Peripheral nerve surgery: Neurosurgery beyond technology

**DOI:** 10.3389/fsurg.2022.1094373

**Published:** 2022-12-06

**Authors:** Lukas Rasulić

**Affiliations:** ^1^ Faculty of Medicine, University of Belgrade, Belgrade, Serbia; ^2^ Clinic for Neurosurgery, Clinical Center of Serbia, Belgrade, Serbia

**Keywords:** peripheral nerve, surgery, technology, functional restoration, quality of life

**Editorial on the Research Topic** Peripheral nerve surgery: Neurosurgery beyond technology

Neurosurgery is a relatively young specialty, and it is heavily riding on the wheels of technology for the past few decades. Both surgeons and staff has shown great understanding and involved all kinds of technological advances, in visualization, imaging, navigation, monitoring etc … . Peripheral nerve surgery, as an integral and indispensable part of neurosurgery has undergone significant development over the past 50 years, primarily with the introduction of microscope and microsurgical techniques and it is taking advantage of improved preoperative and intraoperative diagnostic procedures. Still, there are injuries and diseases which pathophysiology is not yet sufficiently understood, and these complex patients are multidisciplinary challenges. The developments force constant re-evaluation of the techniques and outcomes, and new trends in peripheral nerve surgery represent the advances from a variety of specialties (1).

Solving controversies and dilemmas in peripheral nerve surgery depends on the surgeon, his choice of methods, and his experience, but the main point is that the treatment should not be worse than the disease itself. In recent years we have been relying on the essence of nerve surgery hidden in the acronym—KISS (keep it simple surgeon!). The history and clinical examination remain the foundations of diagnosis in peripheral nerve surgery, however preoperative confirmation using a variety of diagnostic procedures is mandatory. The indications for surgery should be kept clear, avoiding complications, but accepting and dealing with them, when they occur (2).

Basic nerve surgery can be done with simple, basic instruments. Nevertheless, advances in understanding nerve fiber regeneration and possible factors that affect regeneration and improve outcomes, as well as the technological innovations have greatly expanded the indications and improved the results (3).

This Research Topic offers a modern comprehensive approach to peripheral nervous system surgery and consists of nine original research articles and one review paper.

## Basic research

The study by Rochkind S. et al. investigated the innovative Guiding regenerative gel and antigliotic guiding regenerative gel fillings for nerve conduits, prepared with FDA approved agents, and expected to provide an alternative to an autologous nerve graft and enabling reconnecting massive nerve gaps in rabbit model of chronic peripheral nerve injury with massive loss defect that simulates the human condition of chronic injury with large gap. They concluded that the application of and antigliotic guiding regenerative gel led to a stronger nerve recovery and may be an alternative to autologous grafts Rochkind et al. (2021).

Dehu Tian group used an electrospun poly-e-caprolactone-amnion nanofibrous membrane comprising an amnion membrane and nonwoven electrospun poly-e-caprolactone to wrap the sciatic nerve repair site in the rat model of a sciatic nerve transection, and noted effective improvement of nerve regeneration through promotion of Schwann cell proliferation, axon regeneration, limiting muscle denervation and fibrosis after nerve repair, leading to the improved functional recovery Bai et al. (2022).

Another paper from my group, by Lepić et al. reviews the potential role of the diet, nutrients, and supplementation for the outcome augmentation in surgical treatment of peripheral nerve injuries. It emphasizes the importance of standardized diet, micro- and macronutrients intake, and supplementation protocols within the multidisciplinary approach to achieve best possible results and improve nerve regeneration and functional recovery Lepić et al. (2022).

## Clinical evaluation

The aim of the study by Dazhi Yang group from was to compare the clinical characteristics of diabetic carpal tunnel syndrome between 276 patients with neuropathic and non-neuropathic pain The light touch, electrophysiological test results, and psychological factors were found to be related to the neuropathic pain occurrence, found in the majority of patients with diabetic carpal tunnel syndrome Liu et al. (2022).

## Advanced preoperative planning

The pre-spinal route of contralateral C7 nerve transfer developed by Prof. Wendong Xu helps realize the direct anastomosis of the bilateral C7 nerves. However, there are still no less than 20% operations requiring nerve graft, which leads to unfavorable prognosis. This study aimed to explore the optimized pre-spinal route with MRI to further improve the prognosis. According to these data, the middle route was optimally applied to 50 patients, where the rate of nerve transplantation was only 4%, and no such serious complications as vertebral artery and brachial plexus injury occurred. Conclusion According to the 50 patients' low rate of nerve transplantation and their absence of serious complications, the middle route was the optimal Zhao et al. (2022).

## Contemporary surgical management

The two papers from my group are focused on the radial nerve injuries associated with humeral shaft fractures. These lesions are a great burden to everyone involved: the patient, the orthopedic surgeon, work and economical, as well as the social status and institutions, and last but not least the neurosurgeon. The first paper focuses on the etiology, epidemiology and characteristics of patients suffering to these injuries with the findings confirming the previous claim, as the patients are young working population, and the treatment is a great challenge, as presented in the second paper (Rasulić et al. (2022), Rasulić et al. (2021)). The results of surgical management remain diverse. In this paper we presented the outcomes and analyzed the patient, clinical, and surgical procedure related characteristics and factors that may influence the outcome overall.

## New tehnologies

The paper by Qiangqiang Liu of Jiwen Xu group presented a novel minimally invasive robot-assisted percutaneous balloon compression technique for trigeminal neuralgia with short term outcomes in six consecutive patients. Despite requiring a longer time for preoperative preparation, robot-assisted technique allowed for a high degree of accuracy and safety, shortening the learning curve for surgeons unfamiliar with the technique, calling for the further research and development of the percutaneous balloon compression technique as a viable treatment option for trigeminal neuralgia Liu et al. (2022).

## Outcome and quality of life

Quality of life and even the functional recovery of the injured lower extremity nerves are rarely evaluated and the results of our study on peroneal nerve found an apparent advantage of neurolysis, over nerve repair, over tendon transfer procedure, diminishes when all aspects of quality of life are considered, emphasizing individual approach to achieve optimal results in all groups of patients Rasulić et al. (2022).

## Conclusion

As multidisciplinary as the peripheral nerve surgery is, I hope that this Research topic will be helpful to neurosurgeons, vascular and plastic surgeons, anesthesiologists, neurologists, radiologists, neurophysiologists, as well as all physicians involved in the diagnosis and treatment of patients with injuries and diseases of peripheral nerves.

When this Research Topic was proposed, I had a vision to present a broad collection of papers covering all aspects of peripheral nerve surgery, with an insight into modern knowledge about different problems and pathologies, with particular emphasis on the practical understanding of functional recovery, in the light of technology and technological advances, emphasizing the role of surgery beyond technology.
